# Supplementary material for: Genomic and Immunologic Correlates in Prostate Cancer with High Expression of KLK2
Source: Int J Mol Sci. 2024 Feb 13;25(4):2222. doi: 10.3390/ijms25042222 (PMC10889228; doi:10.3390/ijms25042222)
Supplement: Supplementary file 1 [file ijms-25-02222-s001.zip › Supplementary figures.pdf]

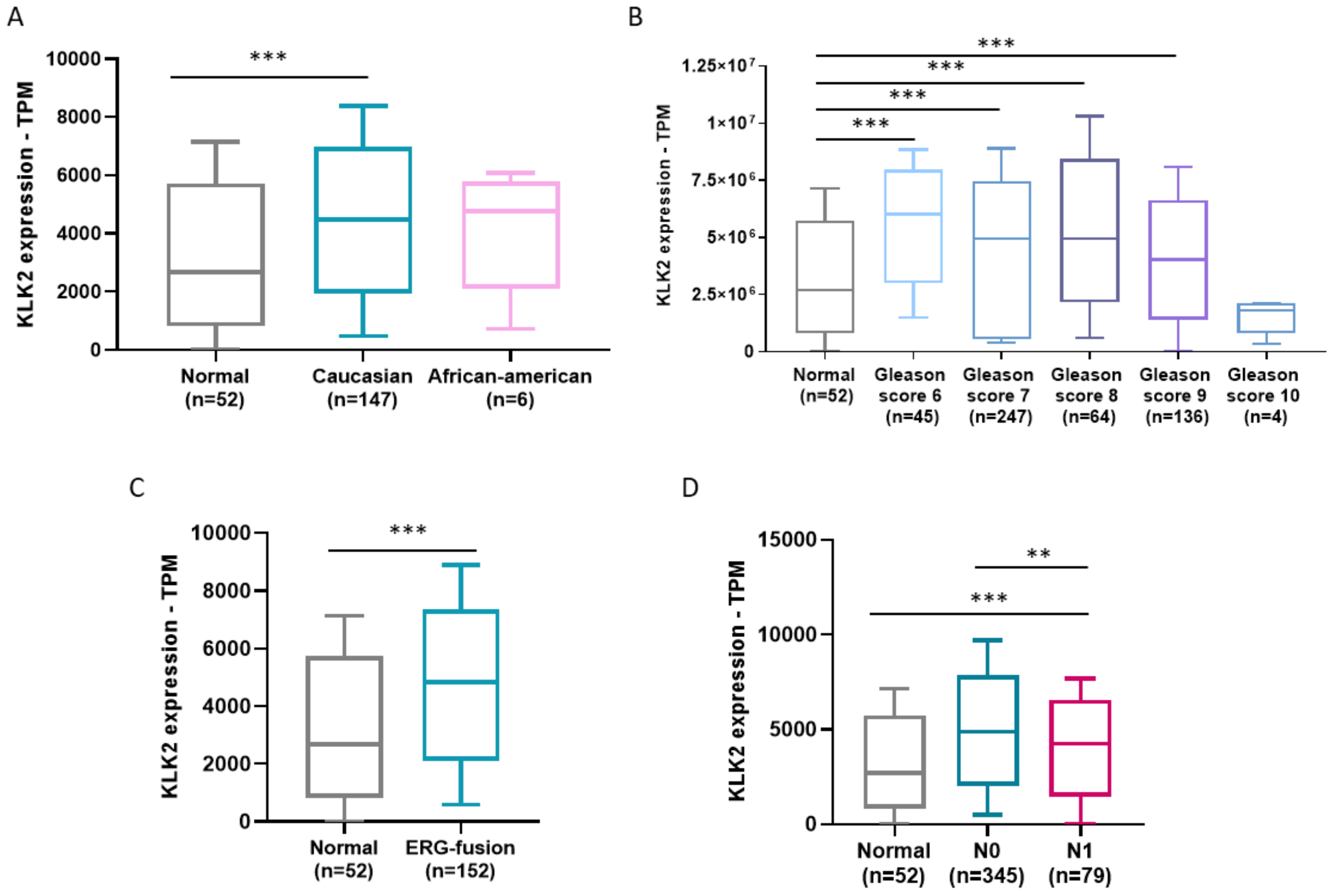

Figure S1. KLK2 expression profile in prostate adenocarcinoma. (A) KLK2 expression levels in normal tissue, caucasian and african-american PRAD patients. (B) Correlation between the transcriptional expression level of KLK2 with the Gleason score, (c) the presence of TMPSSR2-ERG fusion gen and (D) nodal metastasis status in PRAD patients. (N0) no regional lymph node metastasis; (N1) metastases in lymph nodes. Student's t-test was used for statistical tests between groups, (**) indicates a significant difference with $p < 0.01$; (***) $p < 0.001$.

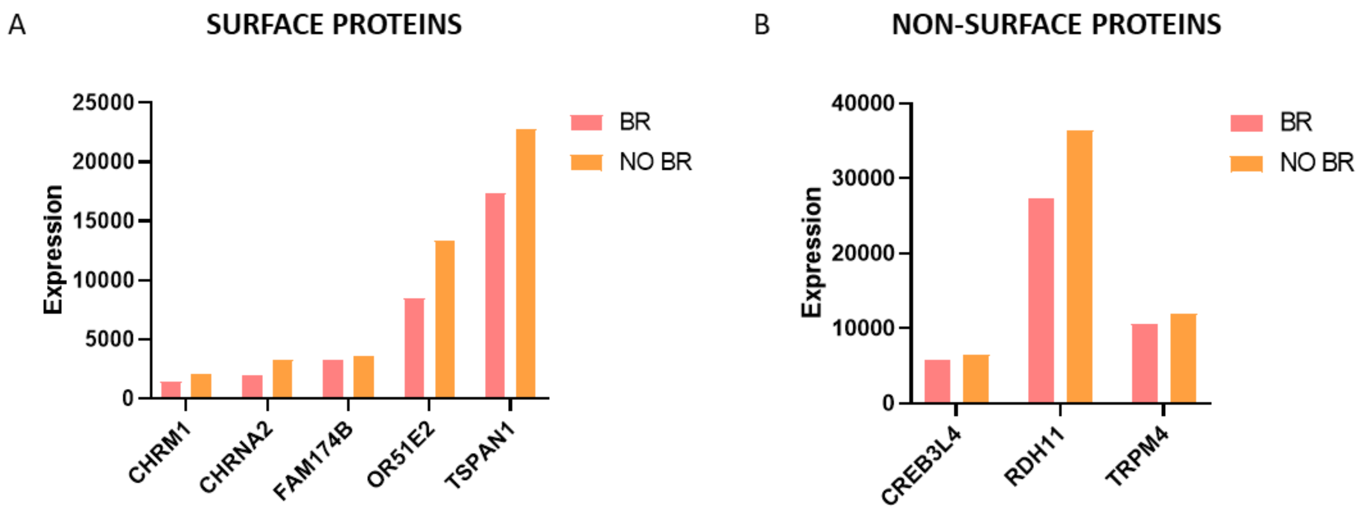

Figure S2. Association of KLK2 and co-upregulated genes with biochemical relapse. Expression levels of surface (A) and non-surface (B) proteins in patients who had suffered o not biochemical recurrence.

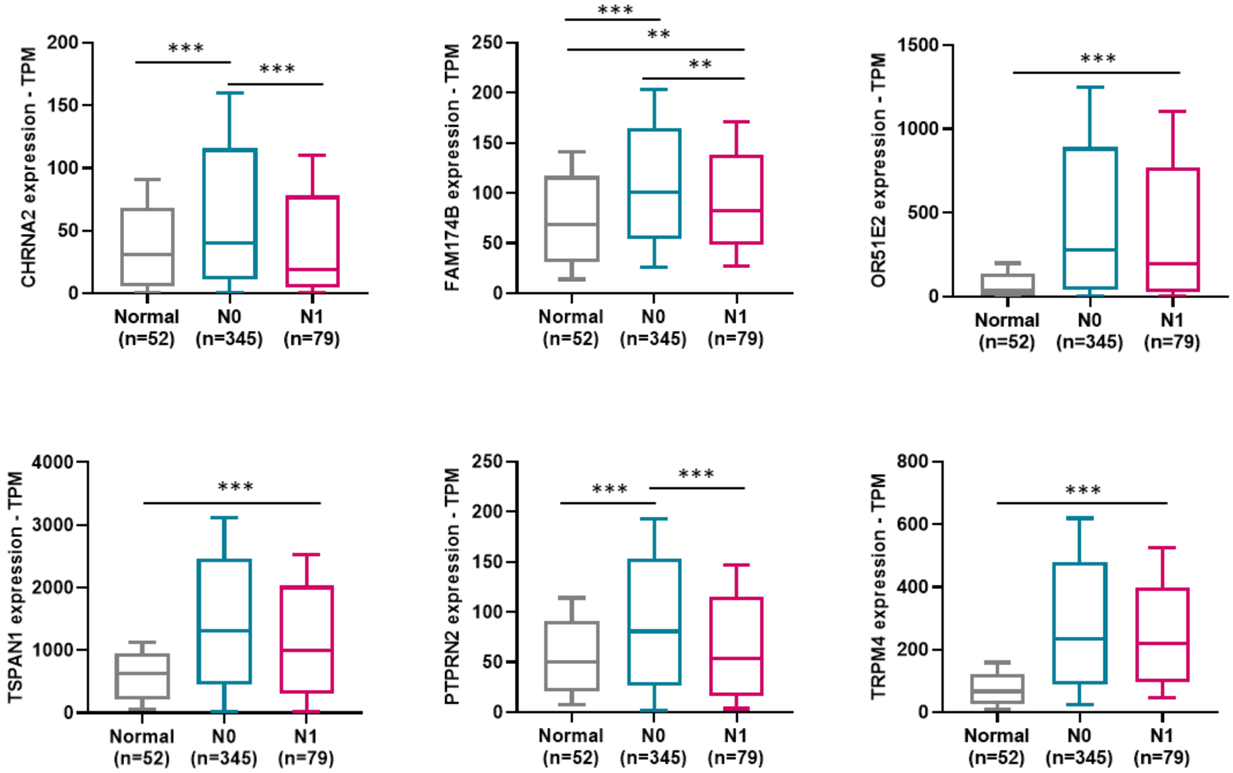

Figure S3. Surface and non-surface proteins expression levels in PRAD based on nodal metastasis status. (N0) no regional lymph node metastasis; (N1) metastases in lymph nodes. Student's t-test was used for statistical tests between groups, (**) indicates a significant difference with $p < 0.01$; (***) $p < 0.001$.

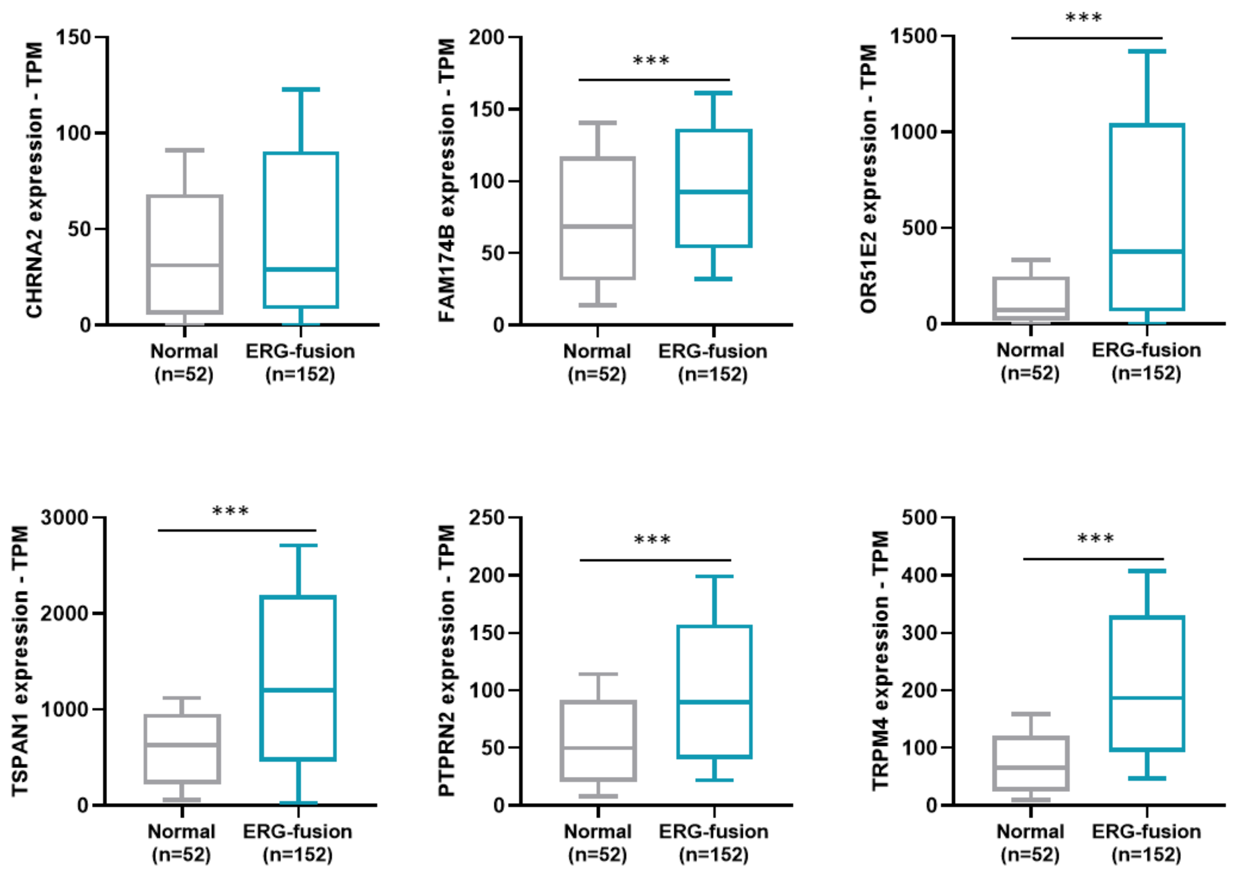

Figure S4. Surface and non-surface proteins expression levels in PRAD tumors with the presence of TMPRSS2-ERG fusion gen and normal tissue. Student's t-test was used for statistical tests between groups, (***) indicates a significant difference with $p < 0.001$.
