# Supplementary material for: Genomic and Immunologic Correlates in Prostate Cancer with High Expression of KLK2
Source: Int J Mol Sci. 2024 Feb 13;25(4):2222. doi: 10.3390/ijms25042222 (PMC10889228; doi:10.3390/ijms25042222)
Supplement: Supplementary file 1 [file ijms-25-02222-s001.zip › Table 1.pdf]

Table S1. Gene expression levels between the high and low KLK2 expression cohorts, and cell location. Cell location is represented with Surface (S), non-surface (NS) and no reported (-).

Gene_name	Mean_exp_KLK2 high	Mean_exp_KLK2 low	FC (res/nonresp)	P-value	Cell location
**CYP4F2**	236.16	63.28	3.7	1.49E-05	-
**ETV4**	1708.41	495.04	3.5	1.16E-03	-
**TFF3**	10985.45	3784.44	2.9	5.63E-12	-
**SERHL2**	489.6	185.28	2.6	1.06E-05	-
**SPON2**	49510.82	20852.94	2.4	1.57E-15	-
**ANPEP**	13836.57	5866.88	2.4	2.64E-09	S
**GNMT**	1779.02	774.93	2.3	5.36E-15	-
**KLK12**	554.37	246.31	2.3	4.82E-04	-
**MESP1**	2085.51	935.16	2.2	1.90E-22	-
**PCGEM1**	1154.38	528.22	2.2	1.58E-10	-
**PCAT4**	1966.45	903.5	2.2	1.37E-09	NS
**LINC01088**	724.54	327.14	2.2	6.12E-09	-
**KCNH6**	724.43	342.71	2.1	1.81E-15	NS
**LOC101929572**	203.27	98.8	2.1	1.16E-09	-
**PCOTH**	304.99	142.93	2.1	2.96E-08	-
**MT1L**	216.9	101.63	2.1	3.37E-06	-
**KLK2**	200614.7	101811.89	2	1.47E-82	-
**LRRC26**	1830.67	923.98	2	1.25E-16	-
**LOC101927870**	147.59	72.83	2	1.23E-11	-
**LOC440910**	268.56	132.96	2	4.01E-10	-
**PEBP4**	1004.97	508.3	2	7.68E-10	-
**C15orf48**	2090.8	1020.24	2	1.07E-09	-
**CCK**	238.5	118.61	2	5.88E-05	-
**KLK3**	616342.6	324258.12	1.9	1.80E-47	-
**SERHL**	188.02	100.43	1.9	7.14E-16	-
**LOC100508046**	274.04	144.5	1.9	2.72E-11	NS
**ARLNC1**	2137.73	1140.24	1.9	2.44E-09	-
**CCDC169**	143.66	76.87	1.9	2.10E-06	-
**CD38**	1700.69	910.38	1.9	2.70E-05	S
**H2AJ**	15581.52	8593.81	1.8	2.25E-21	-
**TMEM220**	1079.58	591.53	1.8	2.75E-16	NS
**CHRNA2**	4495.06	2449.39	1.8	5.43E-14	S
**TMEM220-AS1**	157.8	89.6	1.8	9.27E-12	-
**TMEFF2**	11789.94	6553.59	1.8	1.53E-09	S
**NCAPD3**	14168.52	8039.07	1.8	9.44E-09	-
**GLB1L3**	1387.36	784.68	1.8	1.81E-08	NS
**SYCE1L**	219.09	119.69	1.8	1.09E-07	-
**PRIM2**	1969.87	1123.59	1.8	1.48E-07	-
**MT1M**	560.68	311.53	1.8	3.01E-07	-
**LINC00668**	1459.83	832	1.8	3.39E-05	-

**SCGB1D2**	156.17	87.3	1.8	2.84E-04	-
**TG**	621.86	344.61	1.8	4.55E-03	-
**TRG-AS1**	251.2	140.47	1.8	1.04E-02	-
**SHISA9**	217.32	119.33	1.8	2.69E-02	S
**DSCAM-AS1**	185.11	100.22	1.8	1.89E-01	-
**SLC45A3**	59796.59	35877.67	1.7	1.27E-20	-
**LINC02688**	216.55	129.86	1.7	2.78E-16	-
**ANO7**	5656.58	3264.29	1.7	7.65E-14	S
**TMEM238**	312.42	187.91	1.7	1.73E-13	NS
**FEV**	611	359.88	1.7	3.78E-13	-
**SMS**	14662.9	8723.17	1.7	5.27E-13	-
**RAMP1**	5656.48	3341.76	1.7	4.34E-12	-
**ARG2**	3611.97	2151.52	1.7	2.90E-11	-
**AZGP1**	37773.89	22784.45	1.7	4.52E-11	-
**ACTL10**	328.59	193.14	1.7	6.78E-11	-
**APELA**	146.37	88.68	1.7	7.13E-11	-
**LPAR3**	1681.96	967.43	1.7	3.09E-09	S
**MT1F**	1154.12	689.99	1.7	4.64E-09	-
**PCA3**	12083.03	7108.88	1.7	1.07E-08	-
**CDC42EP5**	1031.16	602.8	1.7	3.44E-08	-
**MIPEP**	2743.13	1660.61	1.7	1.75E-06	-
**ALOX15B**	8268.26	4924.56	1.7	8.13E-06	-
**RFPL2**	453.61	273.74	1.7	2.73E-05	-
**VSTM2A**	350.65	204.09	1.7	5.79E-03	-
**SCGN**	286.49	166.59	1.7	3.67E-01	-
**ADAMTSL1**	1053.85	607.19	1.7	8.81E-01	-
**RABEP2**	3578.29	2298.97	1.6	2.82E-30	-
**POLN**	152.36	98.19	1.6	2.52E-25	-
**TSPAN1**	31825.4	19658.04	1.6	7.92E-23	S
**DCXR**	9232.3	5612.51	1.6	2.93E-21	-
**LENG9**	876.51	554.98	1.6	1.20E-19	-
**RDH11**	46748.69	28813.32	1.6	2.14E-19	NS
**KLK15**	242.3	150.53	1.6	7.88E-15	-
**GREB1**	6161.86	3890.21	1.6	8.54E-14	NS
**IZUMO4**	197.41	125.49	1.6	1.14E-12	-
**ACOXL**	190.85	122.37	1.6	1.32E-12	-
**CTBP1-AS**	299.67	190.75	1.6	1.06E-11	-
**ACPP**	150193.17	95736.78	1.6	9.90E-11	-
**HSF4**	968.02	609.58	1.6	1.34E-10	-
**LDHD**	622.08	397.21	1.6	2.09E-10	-
**OR51E2**	18160.31	11628.84	1.6	6.01E-10	S
**GLYATL1**	2112.94	1352.05	1.6	6.98E-10	-
**APOF**	274.66	174.18	1.6	9.30E-10	-
**ACADL**	871.37	560.92	1.6	1.42E-09	-

**LMAN1L**	271.34	169.61	1.6	3.44E-08	NS
**FAM3B**	4226.7	2702.64	1.6	4.43E-08	-
**C2orf72**	2158.02	1362.5	1.6	1.16E-07	-
**TGM3**	474.07	304.53	1.6	1.30E-06	-
**MT1G**	2316.21	1408.05	1.6	2.63E-06	-
**TENM1**	846.02	540.14	1.6	5.03E-06	S
**KIF12**	586.03	358	1.6	8.29E-06	-
**PHYHD1**	979.69	596.22	1.6	9.32E-06	-
**SLC7A4**	193.31	118.67	1.6	1.00E-05	S
**ACSM1**	3072.61	1956.59	1.6	9.48E-05	NS
**CACNG4**	1033.43	645.78	1.6	1.27E-04	S
**NPY**	32956.08	20227.41	1.6	2.08E-04	-
**UNC5A**	464.51	289.16	1.6	4.61E-04	S
**MSMB**	49588.15	31187.56	1.6	8.71E-04	-
**KLK11**	4897.63	3072.29	1.6	7.37E-03	-
**P3H2**	2581.39	1645.26	1.6	1.25E-02	-
**CHGA**	1464.23	927.53	1.6	1.52E-01	-
**ABCC11**	457.73	292.35	1.6	7.96E-01	S
**SPDEF**	21142.83	14316.94	1.5	1.54E-31	-
**LOC100506100**	149.2	97.57	1.5	5.22E-25	-
**SYTL1**	4556.7	2983.51	1.5	3.92E-22	-
**CENPX**	2554.26	1740.76	1.5	4.66E-21	-
**KLK4**	26048.35	16820	1.5	7.53E-21	-
**GSTZ1**	1621.87	1118.04	1.5	1.15E-20	-
**ADPRM**	643.35	435.79	1.5	1.42E-20	-
**FKBP2**	3332.02	2203.7	1.5	2.28E-20	-
**EFCAB12**	346.4	235.76	1.5	7.32E-17	-
**FBXL8**	568.32	375.18	1.5	9.73E-17	-
**TRPM4**	16087.02	10827.73	1.5	1.87E-16	NS
**SDF2L1**	904.39	617.25	1.5	2.03E-16	-
**MFSD3**	1549.72	1068.3	1.5	6.25E-16	NS
**LINC01137**	448.67	295.86	1.5	9.14E-16	-
**NAA38**	1675.72	1128.36	1.5	2.80E-15	-
**BBS4**	1759.59	1170.4	1.5	5.71E-15	-
**FXYD3**	11207.72	7331.36	1.5	2.23E-14	NS
**UQCC3**	1508.5	1036.78	1.5	1.38E-13	NS
**PAOX**	730.83	491.15	1.5	2.47E-13	NS
**PART1**	2437.26	1626.69	1.5	8.69E-13	-
**HGD**	1980.61	1281.43	1.5	1.69E-12	-
**AZGP1P1**	527.64	351.3	1.5	1.85E-12	-
**CHRM1**	2575.08	1765.84	1.5	2.19E-12	S
**CAMKK2**	11080.79	7581.31	1.5	2.62E-12	-
**SPATC1L**	892.79	595.89	1.5	4.40E-12	-
**RLN1**	243.63	167.13	1.5	5.77E-12	-

**FLJ20021**	2059.37	1328.81	1.5	6.06E-12	-
**REX1BD**	975.94	646.78	1.5	8.55E-12	-
**FBXL15**	566.33	384.74	1.5	4.81E-11	-
**RBP7**	200.95	138.27	1.5	4.72E-10	-
**TRPM8**	16419.79	10896.68	1.5	2.93E-09	-
**GABRG3**	553.68	358.79	1.5	4.51E-09	S
**SULT2B1**	1088.22	719.44	1.5	6.64E-09	-
**RAB27A**	3291.88	2194.39	1.5	1.63E-08	-
**MYBPC1**	7074.26	4569.43	1.5	3.02E-08	-
**SNHG19**	1605	1086.48	1.5	1.29E-07	-
**GADD45G**	3023.81	2038.34	1.5	1.69E-07	-
**PSLNR**	160.13	104.04	1.5	4.46E-07	-
**C3orf14**	471.74	313.13	1.5	1.44E-06	-
**LOC101927495**	133.58	88.87	1.5	2.31E-06	-
**FAAP24**	199.15	131.02	1.5	1.00E-05	-
**OTX1**	263.99	173.63	1.5	5.12E-05	-
**MT1E**	2319.15	1540.5	1.5	5.58E-05	-
**PCSK1N**	341.98	233.59	1.5	5.07E-04	-
**MON1B**	6579.15	4444.36	1.5	8.13E-04	-
**NRG4**	158.58	102.72	1.5	3.17E-03	S
**GSTT2B**	215.24	139.95	1.5	3.71E-03	-
**ARX**	166.3	110.09	1.5	1.17E-02	-
**PRKG2**	342.13	231.03	1.5	5.27E-02	-
**IGSF21**	320.28	220.78	1.5	5.87E-02	-
**ACP5**	1083.66	735.94	1.5	6.60E-02	-
**HSD17B6**	2035.58	1358.22	1.5	8.78E-02	-
**GSTA1**	242.33	166.05	1.5	1.59E-01	-
**RET**	803.92	546.73	1.5	2.19E-01	s
**HPSE2**	513.33	342.7	1.5	5.52E-01	-
**PICK1**	1902.57	1346.78	1.4	3.87E-27	-
**ERP29**	8190.14	5902.28	1.4	3.10E-26	-
**FAM174B**	4771.72	3456.73	1.4	4.15E-26	S
**CREB3L4**	8765.04	6474.4	1.4	3.81E-25	NS
**MYDGF**	4517.23	3128.63	1.4	7.45E-25	-
**NECAB3**	3099.61	2274.08	1.4	1.31E-23	-
**TMEM125**	1927.72	1345.12	1.4	3.77E-23	-
**MPDU1**	2225.91	1579.6	1.4	3.38E-21	NS
**ZNF18**	368.35	270.7	1.4	4.97E-20	-
**BAIAP2**	6029.67	4458.31	1.4	7.62E-20	-
**TSTD1**	2561.58	1884.68	1.4	1.45E-19	-
**KRT18**	22665.55	16567.01	1.4	1.62E-19	-
**EPS8L1**	1375.97	960.64	1.4	2.83E-19	-
**ELMO3**	1565.64	1088.6	1.4	4.09E-19	-
**SERPINB6**	8298.89	6089.08	1.4	1.20E-18	-

**POLD4**	950.37	702.47	1.4	1.45E-18	-
**SYNE4**	1102.26	807.95	1.4	1.61E-18	NS
**CCDC159**	1138.07	828.1	1.4	2.19E-18	-
**HMG20B**	6595.01	4719.35	1.4	2.95E-18	-
**CAMK1**	558.29	403.27	1.4	3.44E-18	-
**NANS**	6063.22	4385.32	1.4	6.80E-18	-
**SSR4**	8652.21	6067.43	1.4	8.36E-18	SN
**GCAT**	1772.07	1259.22	1.4	1.29E-17	-
**RPS19BP1**	2678.66	1920.1	1.4	1.38E-17	-
**NAGLU**	2064.12	1486.02	1.4	2.29E-17	-
**PNPLA7**	2600.92	1896.84	1.4	2.35E-17	NS
**FAAH**	5301.25	3746.09	1.4	4.25E-17	NS
**SELENOO**	1106.1	818.5	1.4	6.06E-17	-
**CHCHD5**	664.89	491.17	1.4	6.94E-17	-
**RAB11B-AS1**	196.24	142.37	1.4	1.33E-16	-
**CRELD2**	1879.87	1388.47	1.4	2.11E-16	-
**ZG16B**	3494.67	2504.16	1.4	7.78E-16	-
**ARSA**	1995.16	1458.17	1.4	7.88E-16	-
**TMEM256**	1426.32	985.93	1.4	8.53E-16	NS
**PEX11G**	270.38	189.79	1.4	2.64E-15	NS
**MPC2**	6580.65	4870.63	1.4	3.66E-15	NS
**SNHG8**	3591.84	2478.91	1.4	3.89E-15	-
**TMEM141**	4856.65	3567.75	1.4	4.55E-15	NS
**DBI**	7771.56	5718.24	1.4	5.64E-15	-
**GLB1L2**	4283.19	3047.55	1.4	7.32E-15	-
**TXNDC17**	1343.24	992	1.4	8.71E-15	-
**CCDC167**	537.97	396.74	1.4	2.19E-14	NS
**DHRS7**	17986.49	12990.58	1.4	2.48E-14	-
**C1orf122**	1428.67	1021.86	1.4	2.49E-14	-
**CHPF**	5778.61	4256.95	1.4	2.83E-14	NS
**EMC9**	603.62	439.59	1.4	2.87E-14	-
**ADPGK**	2566.19	1887.15	1.4	4.20E-14	-
**RAB6C-AS1**	291.34	209.69	1.4	5.07E-14	-
**TXNRD2**	2604.49	1897.24	1.4	5.79E-14	-
**NSMCE1**	3500.78	2499.97	1.4	7.48E-14	-
**LOC100505774**	225.98	159.64	1.4	7.86E-14	-
**PLA2G4F**	1337.39	926.41	1.4	8.18E-14	-
**PPFIA3**	734.21	517.23	1.4	1.03E-13	-
**HOXA11-AS**	1840.95	1341.62	1.4	1.08E-13	-
**HPN**	7946.14	5494.66	1.4	1.13E-13	-
**MTFP1**	169.17	119.8	1.4	1.46E-13	-
**TFPT**	1152.99	810.44	1.4	2.70E-13	-
**MRPL54**	1336.8	954.52	1.4	3.26E-13	-
**MYL5**	660.46	464.35	1.4	9.54E-13	-

**MRPL41**	2940.83	2083.37	1.4	1.46E-12	-
**ANKRD24**	209.82	150.42	1.4	1.49E-12	-
**SEC14L2**	2906.66	2041.96	1.4	2.07E-12	-
**MISP3**	360.45	252.13	1.4	2.13E-12	-
**PET100**	883.43	639.51	1.4	2.44E-12	NS
**PCCA-DT**	740.31	531.22	1.4	2.52E-12	-
**GMPR**	3683.53	2636.39	1.4	2.54E-12	-
**AQP11**	331.78	239.62	1.4	3.21E-12	-
**MIF**	1819.28	1335.59	1.4	3.57E-12	-
**TMPRSS2**	42481.8	30837.02	1.4	3.88E-12	-
**TST**	1445.97	1030.41	1.4	5.80E-12	-
**ALKBH7**	2066.41	1486.48	1.4	1.02E-11	-
**C19orf48**	10271.46	7393.65	1.4	1.08E-11	-
**LOC100996842**	292.7	206.3	1.4	1.14E-11	-
**TARP**	11397.54	8090.32	1.4	1.48E-11	-
**CD320**	2408.22	1757.76	1.4	1.74E-11	S
**SCAND1**	2560.32	1793.86	1.4	2.14E-11	-
**C12orf57**	2902.17	2023.72	1.4	3.24E-11	-
**FAM111A-DT**	457.16	315.6	1.4	4.04E-11	-
**SLC13A3**	1134.59	826.91	1.4	6.81E-11	S
**IGFBP2**	9153.27	6335.83	1.4	8.27E-11	-
**TUBB2A**	1145.3	797.02	1.4	8.96E-11	-
**ANKRD37**	660.26	486.69	1.4	1.11E-10	-
**SDSL**	708.41	524.47	1.4	2.18E-10	-
**GAMT**	1479.17	1079.67	1.4	2.72E-10	-
**SMIM1**	157.39	111.08	1.4	3.70E-10	NS
**MZT2A**	1567.59	1146.66	1.4	5.29E-10	-
**TMEM121B**	859.62	614.43	1.4	5.88E-10	-
**NME3**	2869.67	2080.61	1.4	6.52E-10	-
**HEXD**	1253.59	916.74	1.4	6.87E-10	-
**PYROXD2**	599.52	443.59	1.4	7.82E-10	-
**LRMDA**	317.17	227.96	1.4	8.36E-10	-
**SLC27A5**	352.09	259.84	1.4	8.51E-10	NS
**NDUFA13**	1342.43	956.47	1.4	9.39E-10	NS
**ST20**	120.92	89.46	1.4	1.14E-09	-
**LINC01089**	599.24	414.94	1.4	1.61E-09	-
**SPRN**	188.29	133.01	1.4	1.99E-09	S
**MZT2B**	2751.21	2009.79	1.4	3.09E-09	-
**LINC01146**	223.72	156.1	1.4	4.09E-09	-
**PLEKHB1**	1266.96	931.1	1.4	5.92E-09	-
**SOCS2-AS1**	789.89	583.91	1.4	6.12E-09	-
**RANGRF**	471.58	344.95	1.4	1.06E-08	-
**ENDOD1**	6846.54	5003.78	1.4	1.19E-08	-
**C1orf115**	3641.22	2694.99	1.4	1.45E-08	-

**PTPRN2**	5465.97	3967.99	1.4	1.53E-08	S
**ACAD8**	3504.5	2431.38	1.4	2.84E-08	-
**CPE**	15942.57	11403.43	1.4	4.12E-08	-
**NUDT8**	1206.18	836.79	1.4	4.19E-08	-
**GCNT2**	1935.68	1428.29	1.4	1.44E-07	NS
**SP5**	289.32	207.55	1.4	1.46E-07	-
**SLC23A1**	354.61	262.03	1.4	1.61E-07	S
**SNHG11**	661.77	476.03	1.4	1.65E-07	-
**MRPL12**	911.83	667.51	1.4	2.54E-07	-
**DMRTA1**	298.4	219.79	1.4	5.63E-07	-
**SEMA4G**	885.95	652.74	1.4	9.95E-07	S
**GSTO2**	810.63	599.88	1.4	1.49E-06	-
**FAM13C**	657.09	467.36	1.4	2.02E-06	-
**DLGAP1**	410.07	297.73	1.4	2.42E-06	-
**FGFRL1**	2567.99	1897	1.4	2.81E-06	S
**IRX4**	539.59	392.32	1.4	4.31E-06	-
**PCAT18**	637.79	466.51	1.4	7.11E-06	-
**LAT2**	518.76	360.2	1.4	7.32E-06	NS
**DPY19L2**	351.84	247.64	1.4	1.06E-05	NS
**RRP7BP**	261.09	188.46	1.4	2.27E-05	-
**CYP2J2**	527.26	383.11	1.4	3.48E-05	-
**LOC283177**	518.82	366.3	1.4	4.20E-05	-
**PLIN5**	421.2	301	1.4	4.74E-05	-
**AFF3**	2573.73	1881.08	1.4	5.17E-05	-
**COL28A1**	290.33	209.44	1.4	9.53E-05	-
**FABP5**	1206.69	886.67	1.4	1.18E-04	-
**BRINP3**	332.86	241.59	1.4	1.32E-04	-
**ELFN2**	638.12	467.86	1.4	2.10E-04	S
**SMIM22**	1218.84	900.43	1.4	5.89E-04	NS
**H4-16**	194.5	138.75	1.4	6.24E-04	-
**NPIPB15**	128.72	91.9	1.4	7.22E-04	-
**ORM2**	554.69	403.29	1.4	8.55E-04	-
**COL9A2**	7873.91	5628.98	1.4	6.58E-03	-
**MATK**	298.08	219.2	1.4	7.91E-03	-
**BANK1**	1185.77	846.86	1.4	1.55E-02	-
**SRARP**	543.07	399.97	1.4	2.01E-02	-
**IGF1**	373.1	262.96	1.4	2.94E-02	-
**ZNF385C**	128.46	94.02	1.4	5.76E-02	-
**PTN**	2130.16	1511.02	1.4	1.28E-01	-
**DAB1**	151.42	104.49	1.4	1.69E-01	-
**KL**	830.22	613.21	1.4	3.75E-01	S
**ADAM7**	127.63	93.45	1.4	6.06E-01	S
